# Incidence, preventability, and causality of adverse drug reactions at a university hospital emergency department

**DOI:** 10.1007/s00228-020-03043-3

**Published:** 2020-11-13

**Authors:** Mirjam Kauppila, Janne T. Backman, Mikko Niemi, Outi Lapatto-Reiniluoto

**Affiliations:** 1 Department of Clinical Pharmacology, University of Helsinki and Helsinki University Hospital, Helsinki, Finland; 2 Individualized Drug Therapy Research Program, Faculty of Medicine, University of Helsinki, Helsinki, Finland

**Keywords:** Adverse drug reaction, Antithrombotics, Cytostatics, Preventability, Pharmacogenetics

## Abstract

**Purpose:**

To investigate the characteristics of ADRs in patients admitting at the emergency room of a tertiary hospital.

**Methods:**

We collected the patient records of 1600 emergency room visits of a university hospital in 2018. The patient files were studied retrospectively and all possible ADRs were identified and registered. Patient characteristics, drugs associated with ADRs, causality, severity, preventability, and the role of pharmacogenetics were assessed.

**Results:**

There were 125 cases with ADRs, resulting in a 7.8% overall incidence among emergency visits. The incidence was greatest in visits among elderly patients, reaching 14% (men) to 19% (women) in the 80–89 years age group. The most common causative drugs were warfarin, acetylsalicylic acid (ASA), apixaban, and docetaxel, and the most common ADRs were bleedings and neutropenia and/or severe infections. Only two of the cases might have been prevented by pharmacogenetic testing, as advised in Clinical Pharmacogenetics Implementation Consortium (CPIC) guidelines.

**Conclusion:**

The same ATC classes, antithrombotics and cytostatics, were involved in ADRs causing university clinic hospitalizations as those identified previously in drug-related hospital fatalities. It seems difficult to prevent these events totally, as the treatments are vitally important and their risk-benefit-relationships have been considered thoroughly, and as pharmacogenetic testing could have been useful in only few cases.

**Supplementary Information:**

The online version contains supplementary material available at 10.1007/s00228-020-03043-3.

## Introduction

Successful drug treatment is an obvious goal for healthcare professionals. It improves the prognosis of patient’s life and decreases healthcare costs. Modern medications are very effective, but all of them have adverse effects, too. To avoid adverse drug reactions (ADRs), it is important that drug–drug interactions, contraindications, liver or kidney insufficiency, and other restrictions are considered carefully. For some medications it is, however, particularly complex to balance their benefits and risks for each patient. With effective treatments having narrow therapeutic index, we often have to take risks of causing ADRs.

ADRs are known to cause serious health problems and even deaths in every health care setting [1–3]. Frequencies of ADRs range widely between studies from 3.6 to 61% in hospitalized patients [4, 5]. Reasons for the wide variability are related to the study population, study area, type of hospital, and study methods. For similar reasons, there is also a wide variability in the incidence of ADRs as a cause for hospitalization (0.77 to 9.8%) [2, 6, 7]. Elderly people are found to be especially vulnerable in this respect [8–10].

As these risks are well recognized, information regarding adverse effects in various subgroups or settings is found in many studies [1–3, 11–13]. There is, however, no recent study about ADRs in tertiary care. At a university hospital level, risks causing ADRs are usually well-known and avoidable risks should be quite few. Moreover, there is a lot of enthusiasm and expectations concerning the use of pharmacogenetic testing to help the clinicians to select the right drug and dose for each patient [3].Therefore, we evaluated which drugs were related to ADRs in our hospital at the emergency units covering internal medicine, surgery, neurology, and pulmonology during 6 months and what were the ADRs that they caused. Furthermore, we analyzed whether these risks could have been avoided and whether the use of pharmacogenetics would have helped in avoiding any of these cases.

## Material and methods

This was a retrospective, register-based study on emergency room visits in the Helsinki University Hospital (HUCH) during the period July 1 until December 31, 2018. HUCH is a tertiary hospital covering all the specialties in the capital area of Finland. We focused on the emergency room with internal medicine, surgery (excluding orthopedics and plastic surgery), neurology, and pulmonology. There were about 16,500 emergency visits in these specialties during the study period. We randomly selected 10% of the visits (1600) for detailed evaluation.

Two reviewers, one of which was a specialist in internal medicine and clinical pharmacology, first studied the files of these 1600 visits by hand. The potential cases were further analyzed by two other experienced physicians who are also specialists in clinical pharmacology (Suppl). We checked the diagnoses, which were set at the emergency room, medications used by the patients, and symptoms of the patients. Thereafter, we studied the history of that patient both before and after that visit. We analyzed the medication and checked if there was any reexposure during that visit or later. We carefully evaluated whether the symptoms could have been caused by the medication or if there was a nondrug-related explanation. For the ADRs, we used the definition by WHO. The causality of an ADR was assessed with the criteria suggested first by Karch [14] and modified later by Hallas [15]: (1) known ADR or toxic reaction, (2) a reasonable temporal relationship between commencement of drug therapy and onset of adverse reaction, (3) the adverse reaction disappeared upon discontinuation or dose reduction, (4) the symptom or event could not be explained by any other known condition or predisposition of the patient, and (5) the symptoms reappeared upon reexposure, or laboratory tests showed toxic drug levels or drug-induced metabolic disturbances that explained the symptom.

The cases were categorized either “definite causal relationship” (all five criteria must be fulfilled), “probable causal relationship” (criteria 1–4 must be fulfilled), “possible causal relationship” (criteria 1–3 must be fulfilled), or “unlikely/unevaluable causal relationship”. The diagnoses were classified by using the International Classification of Disease 10th Revision (ICD-10, WHO) and drugs were classified by using the Anatomical Therapeutical Chemical (ATC) system. The severity of the ADRs was assessed according to U.S. National Cancer Institute’s Common Terminology Criteria for Adverse Events (CTCAE).

Preventability of the ADRs was assessed by using similar methods as in many previous studies [16–18]. The best practice–based preventability assessment was based on criteria developed by Hallas [15], and it has been modified for use after that [18, 19]. The preventability assessment included a thorough evaluation of whether the drug was prescribed in accordance with treatment protocols and SPCs, whether required therapeutic monitoring or laboratory tests had been performed and whether all patient data (including allergies, other medications etc.) had been checked.

Additionally, we estimated the proportion of the patients that had an ADR, which may be prevented by genotyping in a specific genetic subset, as guided by the Clinical Pharmacogenetics Implementation Consortium (CPIC) guidelines. First, we identified the patients that had an adverse reaction caused by a drug, which is included in any of the CPIC guidelines. Thereafter, we checked if the specific ADR observed is preventable in patients with a certain genetic profile, given the timing of the ADR event in relation to the preceding duration of the causative medication.

The administrative permission for this study was received from Helsinki University Hospital. Ethical review was not needed as the study involved only register data, and there were no contact to the patients. For statistical analysis and for calculating confidence intervals (CI), we used the Wilson method.

## Results

**Fig 1:**
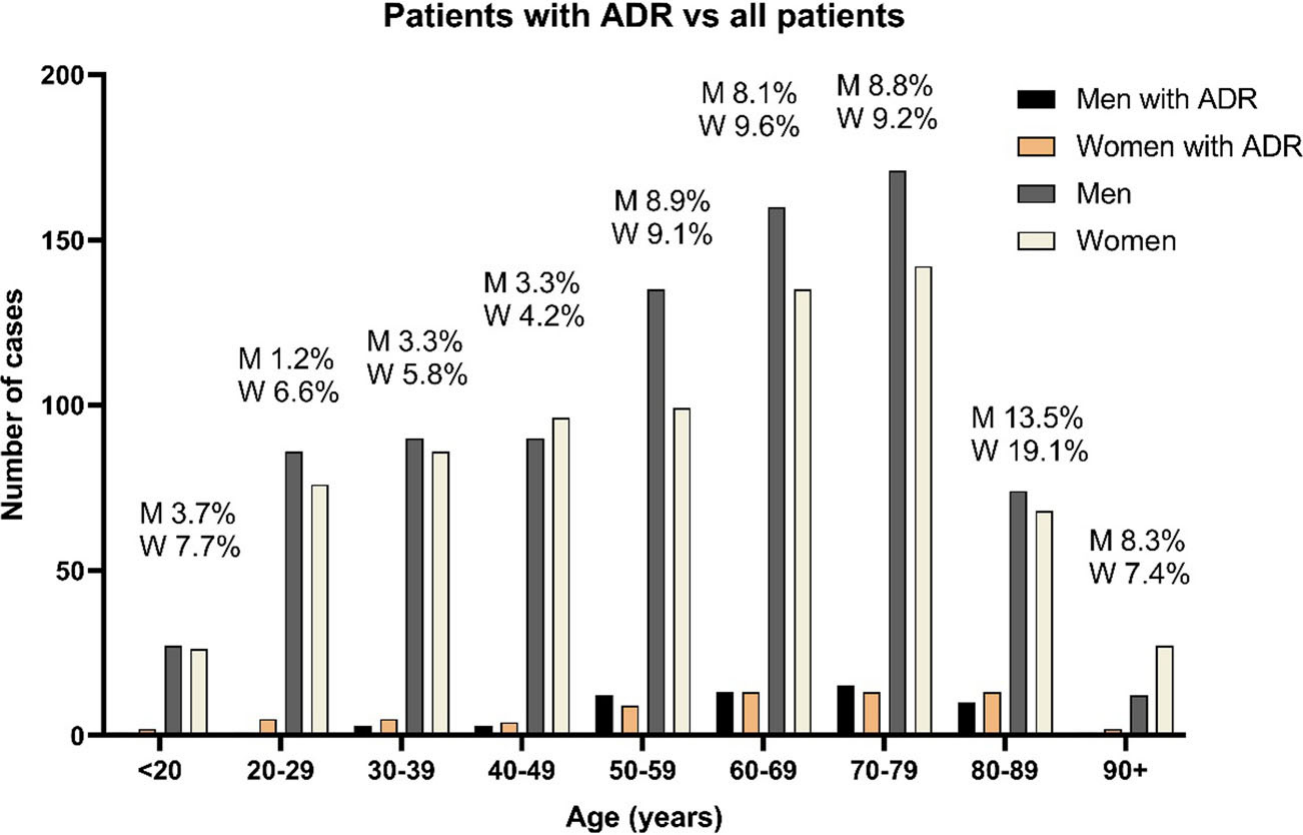
The gender and age specific percentages of ADRs are shown above the bars. M=men, W=women

In our study, there were 1600 emergency visits, of whom 52.8% were men and 47.2% were women. Of these visits, 125 were identified as ADR cases, resulting in a 7.8% incidence. One patient with adalimumab attended ER two times with different ADRs. There were also two patients who had more than one ADR at the time. Thus, the number of ADRs and patients is not the same. Among the ADR cases, the number of women was 66 (52.8%, 95% CI 44.1–61.3%) which was slightly more than that of men (*n* = 59, 47.2%, 95% CI 38.7–55.9%). We compared the percentages of ADRs within each age groups and found that patients aged 80–89 had the highest percentage of ADRs per visits (Fig. 1).

**Fig. 2:**
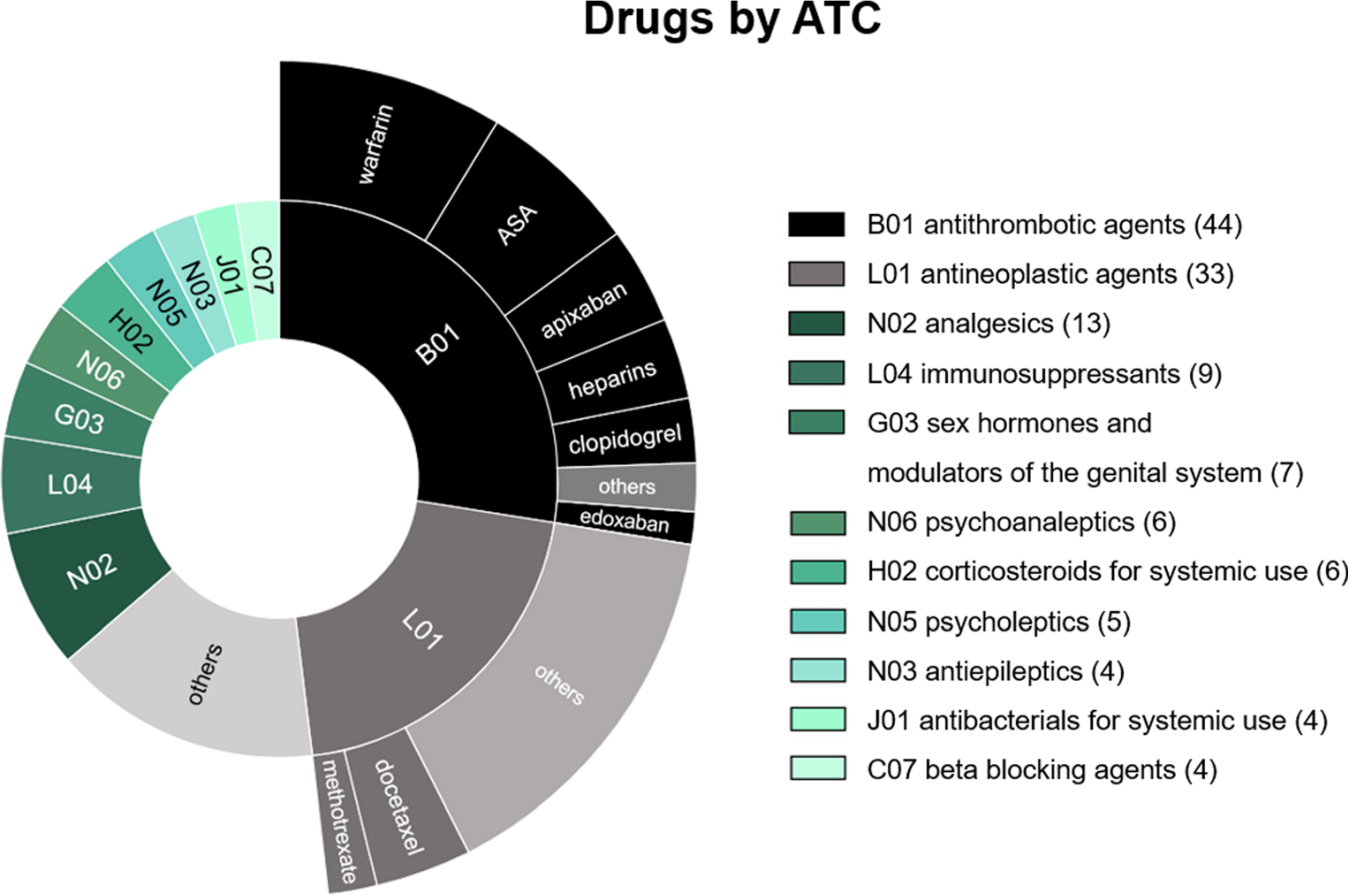
Drugs (*n* = 160) causing ADRs. The ATC-classes causing more than 4 ADRs are shown in the figure

The ATC category most often involved with an ADR was B01 (antithrombotic agents) comprising 27.5% of all cases, followed by L01 (antineoplastic agents, 20.6%). They covered almost half (48.1%) of all ADRs. Opioids (N02) was the third group (8.1%), followed by immunosuppressants (L04; 5.6%). Drugs included in the groups B01 and L01 are specified in Fig. 2. Eleven drugs were involved in three or more cases, of which warfarin and ASA were involved in over 10 cases. More specific list can be found in Suppl.

**Fig. 3:**
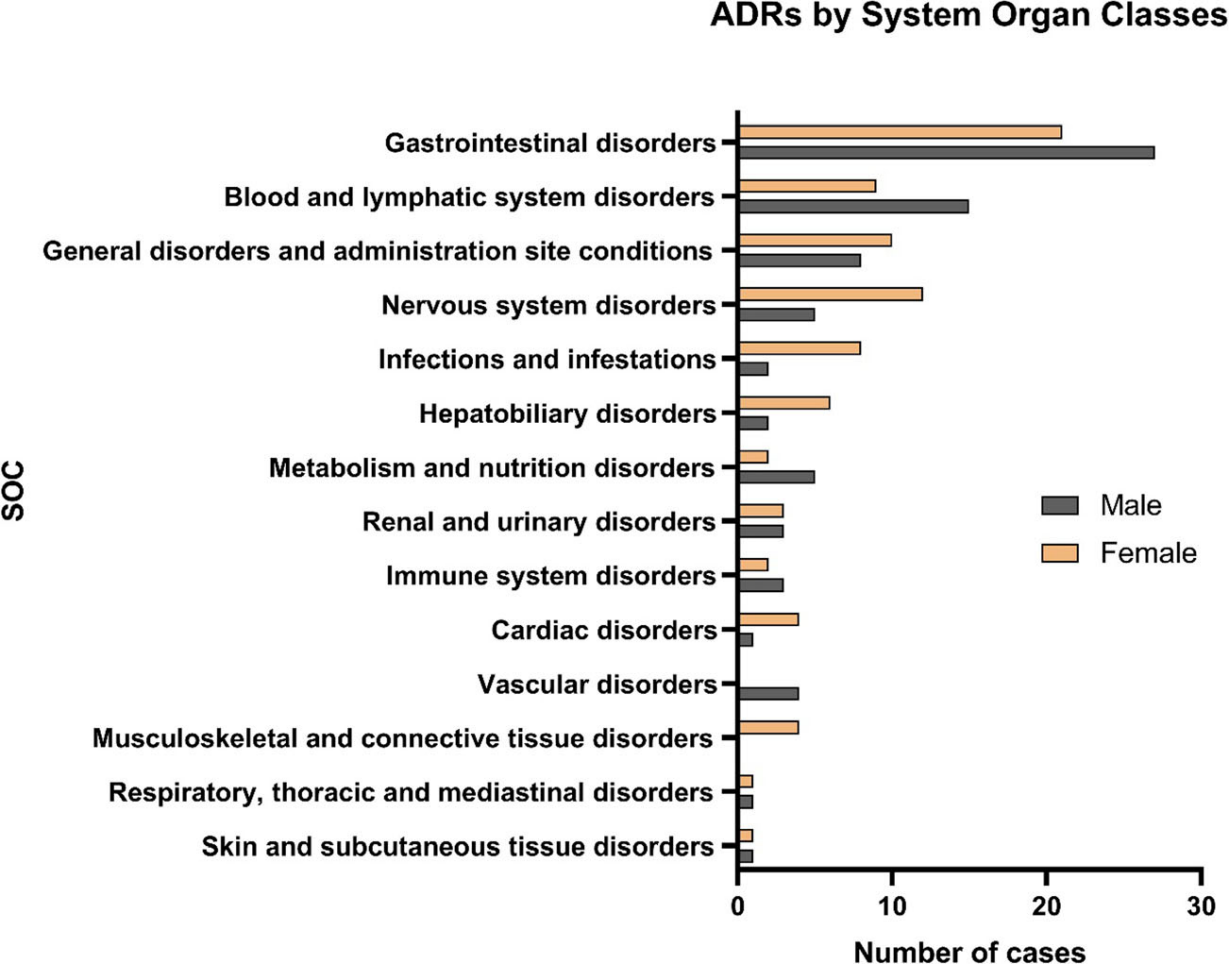
System organ classes involved with ADRs

The most often affected system was gastrointestinal tract (30.0% of ADRs). Blood and lymphatic disorders accounted for 15.0% of the cases, followed by general disorders and administration site disorders (11.3%) and nervous system disorders (10.6%) occurred almost as often. Among ADRs affecting the musculoskeletal system, women were overrepresented. There were four cases with women (100%, 95% CI 51.0–100.0%), whereas men had no musculoskeletal system linked ADRs. Women were overrepresented also with cardiac system disorders (80.0%, 95% CI 37.6–96.4%), infective events (80.0%, 95% CI 49.0–94.3%), and nervous system disorders (70.6%, 95% CI 46.9–86.7%), whereas men were overrepresented in vascular disorders (100%, 95% CI 51.0–100.0%) and metabolic and nutritional disorders (71.4%, 95%CI 35.9–91.8%) (Fig. 3).

Severe ADRs (*n* = 77) outnumbered other classes and together with moderate ADRs (*n* = 56) they comprised 83.1% of the cases. There were only two fatal cases, which were both caused by warfarin, and 15 life-threatening cases. Minority of the cases (*n* = 10) were mild. The causalities of the case were as follows 20.6% definite, 33.8% probable, and 45.6% possible.

In our study, drugs included in CPIC guidelines were involved in 29 cases [20–26]. Two of these cases had an ADR for which a pharmacogenetic test might reduce the risk of that particular ADR. One of the patients was on codeine and had ileus [20]. The other patient was on capecitabine, and he had severe hypokalemia (ad 2.5 mmol/l) [21]. There was another patient with capecitabine too, but his ADR (constipation) could not have been presented by genotyping the patient beforehand. The rest of the cases did not include ADRs, which could have been prevented by a specific genetic test. These drugs were warfarin (14 cases), clopidogrel (4 cases), tacrolimus (2 cases), allopurinol (2 cases), fluorouracil, oxcarbazepine, escitalopram, and citalopram, e.g. warfarin had been in use in every patient for at least 6 months, and genetic testing for warfarin is evaluated to be useful only in the beginning of the treatment.

There were only two cases with a drug-adverse reaction pair which were considered as potentially preventable in a genetic subset by pharmacogenetic testing, according to CPIC guidelines. There were also 27 other ADR cases with drugs mentioned in CPIC guidelines, but the specific ADRs observed were either unrelated to the recommended genotyping or occurred after prolonged treatment (warfarin associated bleedings).

## Discussion

The incidence of ADRs in our university hospital emergency room visits was 7.8%, which was in line with previous studies [2, 27–29]. Comparison to previous studies should be made with caution as study settings, patients, and studied medications differ a lot. The most common drug classes causing ADRs in this study were antithrombotic and antineoplastic agents. They were also most often causing deaths in the same university hospital in earlier studies [6, 30].

In this study, we focused on ADRs in a tertiary hospital emergency ward. ADRs are the most common medication related problem in every health care setting studied [31, 32]. However, most studies focus on primary care setting, while there are only couple of studies from tertiary hospitals [27, 33–36]. There is one recent (2014) study from a university hospital from our country, where the incidence of ADRs was much higher (23.1%) than in our study [37]. Explanation for this difference could be the ages of the patients. They included only patients over 65 years, and the average age was 77. Our patients were 16–94 years, and the average age was 63.4. The characteristics of ADRs depend not only on the studied ward and patient group but also on the country [38, 39]. In a thorough study from India, most ADRs (81.6%) were assessed to be preventable [27]. However, in that study the most common drug classes were antiinfective agents, followed by steroids. Neither of these classes were found in our cases, indicating that there are marked differences in ADR preventability between these two centers. If we exclude these preventable cases from that study, the incidence of the ADRs is close to our study (1.15%).

There are multiple studies evaluating the incidence of ADRs in emergency departments from primary care [13, 28, 40, 41]. Drugs involved in these studies reflect the use of medications in the regional population with its typical age and other patient characteristics. In our Finnish population, children were not included, and the youngest cases were 18–19 years old. While ADRs were observed only infrequently in 20–49 years old people, the majority of ADRs were observed in age groups between 50 and 89 years. In these age groups, more than 8% of the emergency visits were related to ADRs with the highest percentages of 15 to 19% in the 80–89 years age group. There are two likely explanations to this finding: first, many diseases and ADRs are more severe in elderly people, and second, the number of simultaneous medications tends to increase with increasing age [2, 9].

The most important ATC-group in this study was antithrombotic agents (B01) and very close to that was antineoplastic agents (L01). The same two groups were also in the top when we studied ATC-groups involved in fatal cases in the same university hospital area [30]. In that study, cytostatic drugs caused 1.1% of deaths in the hospital and antithrombotic drugs caused 1.0% covering over half of the fatal cases (35/52). In the present study, they covered 48.1% of all ADRs. There were, altogether, 11 drugs causing more than two cases (Table 1), mainly involving either antithrombotics or cytostatics. Antithrombotics (or anticoagulants) and cytostatics have been the most common drugs involved in ADRs also in other studies in every health care setting [2, 3, 9, 10, 16, 28]. Even though the risks connected with these two groups are well known, it is hard to avoid them totally due to their narrow therapeutic window. Both groups are also prescribed to patients with severe diseases, which may also predispose them to ADRs. Deaths caused by cytostatics have diminished year by year in our hospital [30], but at the moment, also fragile patients are treated with them and the medications are more effective meaning that, e.g., leucopenia is inevitable in a subset of patients. There has also been a trend towards an increased intensity of antithrombotic treatments in cardiovascular patients, and overall, the use of antithrombotics has increased, while evaluation of the risk of bleeding has been improved. Perhaps because of improved evaluation and monitoring of patients, there has been even a slight decline in deaths caused by antithrombotic related bleedings during the past decades. In our study, there were six bleeding cases caused by apixaban and 14 by warfarin. Rivaroxaban or edoxaban-related ADRs were not identified in any of the cases. During the same time, the number of users of apixaban and rivaroxaban was about the same in Finland, equaling about 30% of the number of warfarin users. Unfortunately, we were not able to receive the number of the people using these drugs in the specific university hospital area. Nevertheless, it is obvious that the numbers will change in the coming years, after direct anticoagulants have replaced warfarin to a larger extent.

**Table 1:** Drugs involved in three or more cases and the ADRs they caused

Drug	Number of ADRs	Types of ADRs (n)
warfarin	14	Intestinal bleeding (8), hematuria (2), ICH (2), hemarthrosis (1), bruises (1)
ASA	11	Gastrointestinal bleeding (7), bleeding wound (2), hematuria (1), anemia (1)
docetaxel	6	Febrile neutropenia (3), allergic reaction (1), fever (1), erysipelas (1)
apixaban	6	Gastrointestinal bleeding (4), ICH (1), hematuria (1)
clopidogrel	4	Bleeding wound (2), gastrointestinal bleeding (1), anemia (1)
buprenorphine	4	Cholecystitis (2), spasm of neck muscles and migraine (1), headache and disorientation (1)
bisoprolol	4	Bradycardia (3), hypotension (1)
tramadol	3	Acute cholecystitis (1), tremor in lower and upper extremities and anxiousness (1), nausea, sweating and tremor (1)
prednisolone	3	Sepsis and pneumonia (1), infection NUD (1), hyperglycemia (1)
oxycodone	3	Ileus (2), worsening of cancer pain (1)
methotrexate	3	Respiratory tract infection (1), pulmonary insufficiency (1), hepatic cirrhosis (1)
others	99	

Most ADRs were gastrointestinal disorders followed by ADRs involving blood and lymphatic disorders. This is in line with the most often involved ATC classes, antithrombotics, and cytostatics. Furthermore, most ADRs were classified as severe, as many of them were serious bleedings, infections, or other serious toxicities. This finding probably also reflects the university hospital site of the study, as most mild cases are treated in primary care, while more severe cases are usually directed to the university clinic. Clinics involving oncology and hematology patients receiving cytostatics also typically report more severe cases than hospitals without such patients.

There is no universally accepted method for ADR causality grading, although a number of causality assessment scales have been published. Some studies have compared different scales, and they have usually found a poor agreement between the scales [42, 43]. Naranjo criteria have been used in many studies, as well as Hallas criteria. The Hallas criteria include the same general aspects as the Naranjo scale includes, but the Hallas criteria were more suitable for this study [15].

The preventability of ADRs seems to vary a lot between studies, at least from 4.3 to 83% [2, 44, 45]. This variation is not only dependent on different scales but also on different characteristics of patients and drugs. Preventable ADRs include, e.g., those caused by antihypertensives and antibiotics in many studies, while cases assessed as not to be prevented include medications like cytostatics. Most ADRs in our study were caused by antithrombotic agents and antineoplastic agents, which are used only after a precise consideration of the risk-benefit relationship of the treatments that are known to cause ADRs to a small subset of patients. There were, however, nine cases where ADR might have been preventable. Those cases included buprenorphine (2), tramadole (3), oxycodone (2), and bisoprolol (2). In these cases, opioids could have been replaced with other pain medications and bisoprolol could have been used with a lower dose. With these nine cases preventability of ADRs would have been 7.2% of all ADRs.

One aim of this study was to find out if pharmacogenetic testing could have prevented some ADRs. We used the international CPIC guidelines to evaluate if there were recommendations concerning any of the ADR causing drugs. For example, in case of allopurinol, which can be prescribed more safely after testing the HLA-B*5801-allele, the test could only be used to prevent Stevens-Johnson syndrome, not fever or gastrointestinal pain, which were the ADRs of the respective patients [22]. After evaluating every ADR causing drug, we found only two cases for which pharmacogenetic testing might have prevented the ADRs; one patient with hypokalemia related to capecitabine induced diarrhea and one with ileus caused by codeine. For capecitabine, there is evidence that pharmacogenetic testing could prevent severe toxicity [21], and for codeine gastrointestinal opioid adverse effects are shown to be associated with the CYP2D6 metabolizer phenotype [20]. There were also other ADRs caused by drugs included in CPIC guidelines. Warfarin was involved in 14 bleeding cases. Genotype-guided warfarin dosing is thought to be beneficial only when warfarin is started, as it helps to find the first stable international normalized ratio (INR) [23]. However, in all our cases, warfarin had been in use for more than 6 months. Therefore, it was considered unlikely that pharmacogenetic testing could have been used to prevent the ADRs, although carriers of the CYP2C9*3 allele can have a higher risk of bleeding even after prolonged warfarin treatment [46]. Other drugs included in CPIC guidelines were the fluoropyrimidines capecitabine and fluorouracil, whose severe toxicity could be reduced by genotype-guided dosing [21]. However, the cases in this study did not have severe toxicity, but only constipation and fever without neutropenia, and we considered that these ADRs could not have been prevented by pharmacogenetic testing. Further cases included hyponatremia caused by escitalopram or citalopram that we considered not preventable by pharmacogenetic testing [24], as there is no compelling evidence showing that hyponatremia is (es)citalopram concentration-dependent. Yet, there are two cases of escitalopram dose-dependent hyponatremia [47, 48].

## Strengths and limitations

The number of emergency visits in our study was quite large. There were 16,535 ER visits during the six study months, and 1600 of them were randomized to our study. The patient files of these 1600 visits were studied carefully by one student and three experienced clinicians and clinical pharmacologists, and all possible ADRs were assessed and only real ADRs were included (case 1 Suppl).

We did not consider lack of drug effect to be an ADR. Therefore, there might have been cases that potentially could have been prevented by a pharmacogenetic testing. For example, clopidogrel is converted to its active metabolite by CYP2C19, and poor metabolizers with two unfunctional copies of CYP2C19 have reduced amount of active clopidogrel metabolites [25], which may result in blood clotting.

## Conclusion

The same ATC categories and medications are in top when assessed the ADRs causing hospitalizations and fatal cases caused by medications. It seems difficult to prevent these events totally as the treatments are vitally important and risk-benefit-relationship has been considered thoroughly.

## Supplementary Information

ESM 1 (DOCX 157 kb).

## Data Availability

Not applicable.
