# Supplementary material for: Incidence, preventability, and causality of adverse drug reactions at a university hospital emergency department
Source: Eur J Clin Pharmacol. 2020 Nov 13;77(4):643–50. doi: 10.1007/s00228-020-03043-3 (PMC7935812; doi:10.1007/s00228-020-03043-3)
Supplement: Supplementary file 1 — (DOCX 157 kb). [file 228_2020_3043_MOESM1_ESM.docx]

Supplementary Document

Flow diagram of search strategy


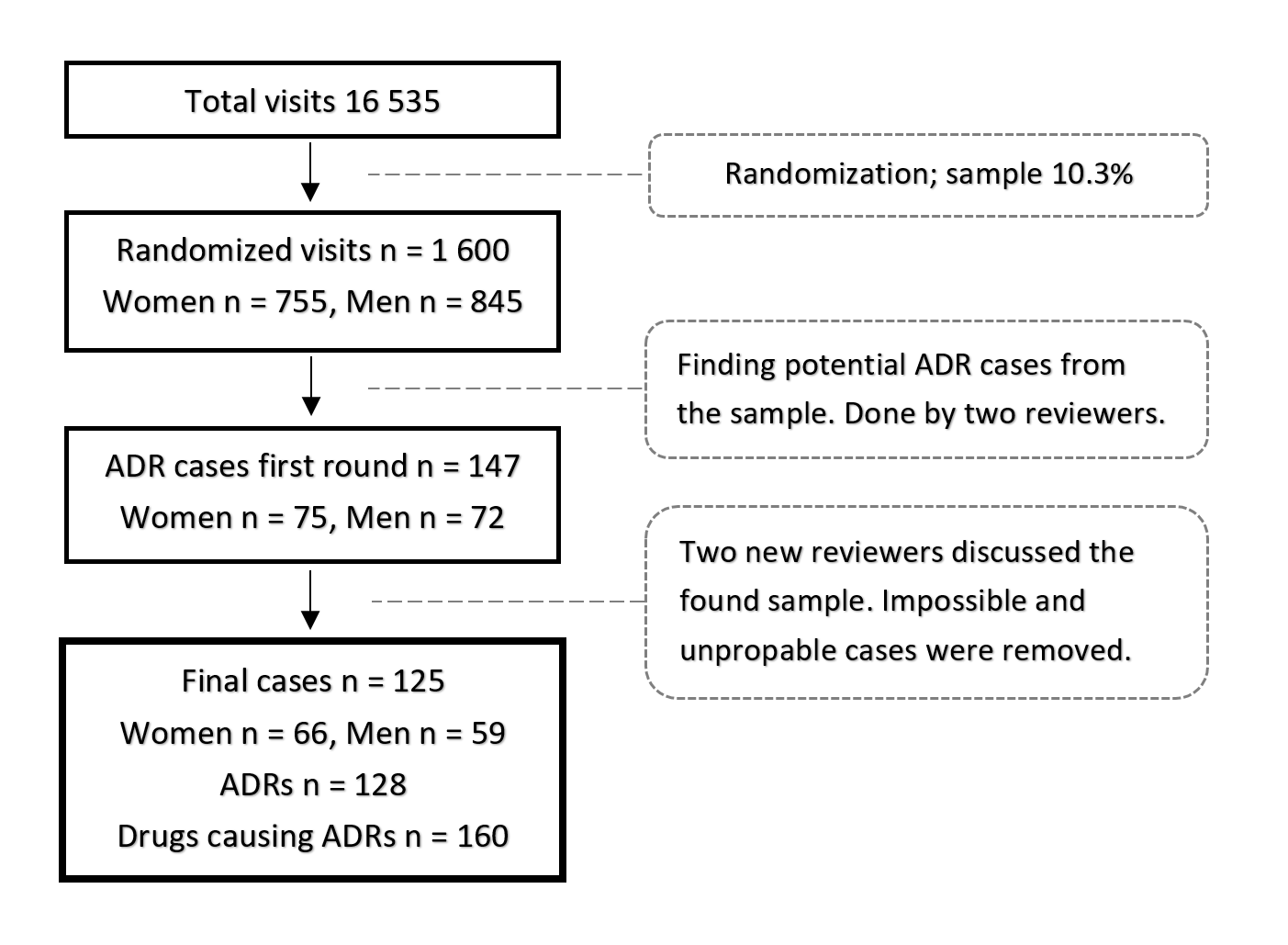


Supplementary Document: Two reviewers, one of which was a specialist in internal medicine and clinical pharmacology, first studied the files of these 1600 visits by hand. Both of them studied all 1600 cases. The potential cases were further analyzed by two other experienced physicians who are also specialists in clinical pharmacology. Time spent to one case differed much during the first round. There were “easy” cases (like a patient being stabbed) which needed just one or two minutes. On the other hand, multimorbid cases with multiple medications needed very thorough studying and some of them took even 30 minutes. The subsequent reviewers discussed the cases together with the first reviewers until consensus was found, approx 10-30 minutes per case.

Here is an example of one case:

**Case 1:**

48-year old male was admitted at the ER with hyponatremia (113 nmol/l) and headache. He was on amitriptyline, while diclofenac and etoricoxib were started couple of weeks ago. At first sight, hyponatremia could have been related to these medications but it turned out that the patient had macroadenoma with hypocortisolism, and following surgical treatment, he was able to use amitriptyline without any problem.

Drugs causing ADRs.

There were 160 drugs causing ADRs. B01 antithrombotic agents (44 ADRs), L01 antineoplastic agents (33), N02 analgesics (13), L04 immunosuppressants (9), G03 sex hormones and modulators of the genital system (7), N06 psychoanaleptics (6), H02 corticosteroids for systemic use (6), N05 psycholeptics (5), N03 antiepileptics (4), J01 antibacterials for systemic use (4), C07 beta blocking agents (4). Drugs causing 2-3 cases: A02 drugs for acid related disorders, A10 drugs used in diabetes, C09 agents acting on the renin-angiotensin system, M01 anti-inflammatory and antirheumatic products. Drugs causing one case: A07 antidiarrheals, intestinal antiinflammatory/antiinfective agents, A12 mineral supplements, C01 cardiac therapy, C02 antihypertensives, C03 diuretics, C08 calcium channel blockers, C10 lipid modifying agents, D10 anti-acne preparations, G04 urologicals, J04 antimycobacterials, L02 endocrine therapy, M04 antigout preparations, M05 drugs for threatment of bone diseases, V08 contrast media.
